# Localisations particulières de l'histiocytose langerhansienne chez l'enfant, scapula et pubis: à propos de deux cas

**DOI:** 10.11604/pamj.2014.18.328.1570

**Published:** 2014-08-25

**Authors:** Karima Atarraf, Lamiae Chater, Mounir Arroud, My Abderrahman Afifi

**Affiliations:** 1 Service de Chirurgie Traumato-orthopédie, Pédiatrique, CHU Hassan II, Faculté de Médecine et de Pharmacie, Université sidi Mohammed ben Abdullah, Fès

**Keywords:** Histiocytose X, scapula, ischion, enfant, histiocytosis X, scapula, ischion, child

## Abstract

L'histiocytose X ou histiocytose de Langerhans est une maladie de l'enfant et de l'adulte jeune. Dont l'incidence est estimée à 1 cas sur 200 000 par an. C'est une maladie au spectre clinique très divers, allant du simple granulome éosinophile à la forme grave multiviscérale avec dysfonctionnement d'organe. Les auteurs rapportent deux observations concernant deux localisations assez rares de cette maladie, au niveau du pubis chez le premier enfant, et au niveau de la scapula chez le deuxième. Chez nos deux malades la localisation était focale, et l’évolution était favorable. A travers ces deux observations, nous allons essayer de décrire les différents aspects cliniques et radiologiques et discuter a travers une revue de littérature les démarches diagnostiques et thérapeutiques de cette maladie rare.

## Introduction

L'histiocytose langerhansienne (HL) est une pathologie rare, d’étiologie inconnue. Elle est due à une prolifération anormale de cellules de Langerhans dans différents tissus et organes (os, peau, ganglions). Ainsi les expressions cliniques sont différentes en fonction de leur extension. Nous rapportons deux cas d'histiocytose localisée au niveau de la scapula et de l'ischion chez deux enfants de 12 ans et 7 ans respectivement. A travers ces deux observations nous allons faire une revue de la littérature.

## Patient et observation

**Figure 1:**
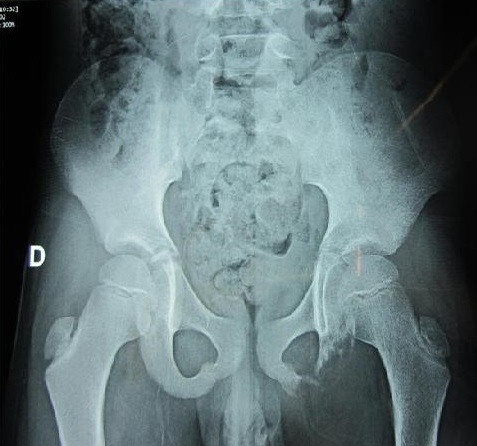
Radiographie du bassin montrant une lésion ostéolytique de l'ischion gauche

**Figure 2:**
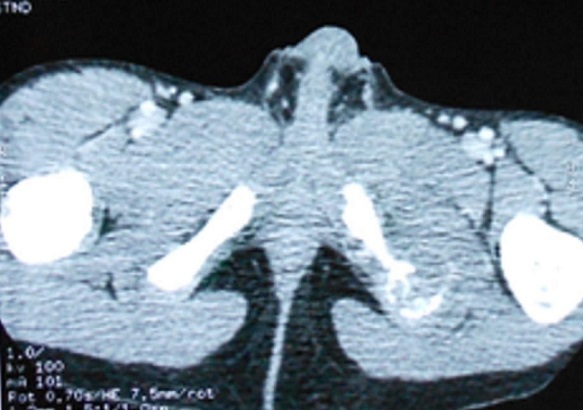
IRM du bassin montrant la lyse osseuse

**Observation n°1:** Enfant de 7 ans, de sexe masculin; admis pour prise en charge d'une douleur périnéale évoluant depuis deux mois; avec installation ultérieure d'une boiterie apyrétique. La radiographie du bassin a trouvé une lésion ostéolytique de la branche ischiatique gauche (Figure 1). L'IRM du pelvis n'a pas objectivé d'extension vers les parties molles (Figure 2). Une biopsie osseuse a été réalisée est revenue en faveur d'une histiocytose X. Le reste du bilan d'extension notamment la scintigraphie osseuse et l’échographie abdominale se sont révélées normales. L'enfant a été mis sous anti inflammatoire non stéroïdien. L’évolution a été bonne sur le plan clinique et radiologique avec reconstruction osseuse manifeste sur un recul de 02 ans.

**Figure 3:**
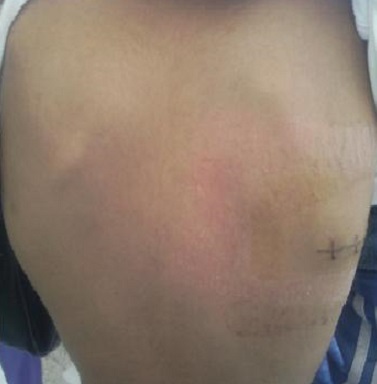
Image montrant une tuméfaction sous scapulaire droite

**Observation n°2:** Enfant âgé de 12 ans, sans antécédents pathologiques particuliers, qui a présenté depuis 2 mois une tuméfaction scapulaire augmentant progressivement de volume et évoluant dans un contexte de conservation de l’état général et d'apyrexie. L'examen clinique a trouvé une tuméfaction en regard de la pointe de la scapula droite de 6cm de grand axe sans signes inflammatoires en regard (Figure 3). La radiographie de la scapula a objectivé une condensation osseuse, le patient a bénéficié d'une biopsie osseuse, qui est revenue en faveur d'une histiocytose X. le malade a été mis sous anti-inflammatoires non stéroïdiens et l’évolution à été marquée surtout par la régression de la douleur. Avec une légère diminution de la masse sur un recul de 03ans.

## Discussion

L'histiocytose X, ou plus précisément l'histiocytose de Langerhans, est définie par une prolifération des cellules du système mononucléaire appelées cellules de Langerhans. Chez l'enfant, l'incidence est estimée à 1 cas sur 200 000 par an [1]. La majorité des cas surviennent dans l'enfance. L'os est l'organe le plus souvent touché (80%), avec prédominance d'atteinte du crane et des os longs chez l'enfant. La localisation au niveau de la scapula et de l'ischion n'a jamais été décrite.

Chez l'enfant, le diagnostic peut être posé à tout âge, de la naissance à l'adolescence, avec un pic de fréquence entre 1 et 3 ans (1), et un sex-ratio de 1/2. L’étiopathogénie de l'histiocytose X reste inconnue. Sa localisation peut être ubiquitaire. Les anomalies osseuses sont les plus fréquentes, 60% des cas rapportés ne touchent que l'os, notamment le crâne, la face et les côtes [2, 3]. Cette affection, qui est diagnostiquée de plus en plus fréquemment, est due à des cellules précurseurs, qui sont les cellules dendritiques (cellules possédant une fonction de stimulation dans la réponse immunitaire). Ces cellules sont retrouvées de façon anormale dans les tissus.

La confirmation diagnostic est apportée par l'analyse histologique mettant en évidence la prolifération caractéristique de cellules porteuses de l'antigène CD1a et de la protéine S100. Le pronostic est indépendant de l’âge et du sexe. Il est souvent considéré comme imprévisible au moment du diagnostic [1]. Les formes graves peuvent évoluer défavorablement en quelques semaines. A l'inverse, un granulome éosinophile peut spontanément régresser ou être suivi plusieurs années plus tard d'autres nouvelles localisations osseuses. Le pronostic dépend de la classification de Lahaye-Osband. Ainsi l’âge, le nombre de localisations initiales, le dysfonctionnement d'organes et la réponse initiale au traitement sont les quatre éléments dont la valeur pronostique est retenue [1, 4].

Le traitement de l'histiocytose de Langerhans reste à l'heure actuelle toujours controversé vu la méconnaissance de sa pathogénie, la rareté des formes graves et la diversité des présentations cliniques et de leur évolution qui expliquent en grande partie la difficulté de prise en charge de ces patients [1, 5, 6].

La prise en charge des formes uni-focales est le plus souvent limitée avec une évolution bénigne. Les localisations osseuses uniques ou peu nombreuses ne nécessitent généralement pas de traitement en dehors de la biopsie pour confirmer le diagnostic et d'un curetage éventuel ou encore d'une injection locale de corticoïdes [7, 8]. La radiothérapie, autrefois largement utilisée, doit être contre-indiquée de principe dans la maladie. Elle ne sera plus utilisée que de manière exceptionnelle dans des lésions menaçant le pronostic fonctionnel.

## Conclusion

L'histiocytose Langhéransienne est une pathologie rare, hétérogène dans sa présentation clinique, avec une évolution imprévisible. L'imagerie des structures osseuses touchées peut évoquer le diagnostic devant une ostéolyse bien limitée, entourée d'une ostéosclérose périphérique, unique ou multiple. II est beaucoup plus difficile devant une localisation unique ou rare nécessitant le recours à l’étude histologique. Le pronostic dépend du nombre et de la topographie des sites affectés et de l’âge du patient.

## References

[1] Brichard B (2000). Histiocytose de langerhans: nouveautés concernant la compréhension d'une maladie énigmatique= Langerhans cell histiocytosis. Louvain med..

[2] Howarth DM, Gilchrist GS, Mullan BP, Wiseman GA, Edmonson JH, Schomberg PJ (1999). Langerhans cell histiocytosis: diagnosis, natural history, management, and outcome. Cancer..

[3] Kilpatrick SE, Wenger DE, Gilchrist GS, Shives TC, Wollan PC, Unni KK (1995). Langerhans’ cell histiocytosis (histiocytosis X) of bone: A clinicopathologic analysis of 263 pediatric and adult cases. Cancer..

[4] Lahey E (1975). Histiocytosis x--an analysis of prognostic factors. J Pediatr..

[5] Arceci RJ, Brenner MK, Pritchard J (1998). Controversies and new approaches to treatment of Langerhans cell histiocytosis. Hematol Oncol Clin North Am..

[6] Broadbent V, Gardner H (1998). Current therapy for Langerhans cell histiocytosis. Hematol Oncol Clin North Am..

[7] Bernstrand C, Björk O, Ahström L, Henter JI (1996). Intralesional steroids in Langerhans cell histiocytosis of bone. Acta Paediatr..

[8] Yasko AW, Fanning CV, Ayala AG, Carrasco CH, Murray JA (1998). Percutaneous techniques for the diagnosis and treatment of localized Langerhans-cell histiocytosis (eosinophilic granuloma of bone). J Bone Joint Surg Am..

